# Closed-loop neuroscience and neuroengineering

**DOI:** 10.3389/fncir.2014.00115

**Published:** 2014-09-23

**Authors:** Steve M. Potter, Ahmed El Hady, Eberhard E. Fetz

**Affiliations:** ^1^Laboratory for Neuroengineering, Coulter Department of Biomedical Engineering, Georgia Institute of TechnologyAtlanta, GA, USA; ^2^Department of Non Linear Dynamics, Max Planck Institute for Dynamics and Self OrganizationGoettingen, Germany; ^3^Departments of Physiology and Biophysics and Bioengineering, Washington National Primate Research Center, University of WashingtonSeattle, WA, USA

**Keywords:** feedback, real-time, BCI, microelectrode array, CMOS, DBS, stimulation

Feedback and closed-loop circuits exist in just about every part of the nervous system. It is curious, therefore, that for decades neuroscientists have been probing the nervous system in an open-loop manner to understand it. Instead of the linear, reductionistic “stimulate → record response” approach, a more modern approach is taking hold: closed-loop neuroscience. It respects the inherent “loopiness” of neural circuits, and the fact that the nervous system is embodied, and embedded in an environment. Through active sensing, behaving animals can influence their environment in ways that alter subsequent sensory inputs. Therefore, loops abound not only in the nervous system itself, but through its dynamic interactions with the world. By interposing our own technology in some of these loops, we can achieve unprecedented control over the system being studied and explore the functional consequences. This Research Topic, “Closing the Loop Around Neural Systems,” presents a diverse set of recent methodological, scientific and theoretical advances from neuroscientists and neuroengineers who are pioneering closed-loop neuroscience.

As shown here, cutting-edge researchers are taking advantage of real-time or “on-line” processing of large streams of neural data. This has become feasible thanks to advances in computer processing power, in electronics such as microprocessors and field-programmable gate arrays (FPGAs), and in specialized and open-source software. These advances have enabled a wide variety of new neuroscience approaches to understanding, modulating, and interfacing with the nervous system—approaches in which the variables being monitored can influence the experiment in progress, just as active sensing can influence an animal's next input.

Our call for submissions to this Frontiers in Neural Circuits Research Topic yielded an overwhelming response, indicating that closing the loop around neural systems is an exciting and rapidly expanding field. Perhaps this is because of the diversity of ways in which “closed-loops” can be interpreted and implemented. This Research Topic presents seven Methods articles, 16 Original Research articles, and seven Reviews, Mini-Reviews, and Perspectives, for a total of 30 accepted papers published in Frontiers in Neural Circuits between April 2012 and October 2013. A map showing the locations of all the contributors^1^ reveals that most are in the USA and Europe, although researchers in Russia, Japan, and Israel are also represented.

Several articles describe or review new technologies that increase the options for closed-loop neuroscience. Two papers by Bareket-Keren and Hanein (2013) and Robinson et al. (2013) review the latest in carbon nanotube and nanowire multi-electrode arrays (MEAs) for neural interfacing. Franke et al. (2012) review high-density MEAs with many electrodes and real-time spike sorting. Müller et al. (2013) present sophisticated hardware and software for very fast (sub-millisecond) closed-loop recording and stimulation of cultured networks using their CMOS array with 11,011 electrodes. Newman et al. (2013) created an application programming interface (API) for their open-source NeuroRighter electrophysiology system that greatly enhances its ability to carry out closed-loop experiments in which recorded signals trigger electrical stimulation or other hardware. Five examples of closed loop experiments *in vitro* and *in vivo* are described.

A number of articles present advances using acute or cultured networks *in vitro*. Bonifazi et al. (2013) present EU Brain Bow project efforts in progress, to create and study bi-directional neural interfaces. Their work includes both patterned dissociated cultures and their responses to laser ablation, and a whole-brain *in vitro* preparation and its response to focal ischemia. The goal is to develop the closed-loop prostheses of the future. Tessadori et al. (2012) present their Hybrain2 software for real-time control of hybrid neural-robotic systems, consisting in this case of a virtual wheeled robot interfaced to a living hippocampal network on an MEA. Brewer et al. (2013) reconstructed a hippocampal trisynaptic loop *in vitro* on an MEA with small tunnels for neurites to grow through. Pimashkin et al. (2013) used an adaptively enhanced learning protocol to study learning in dissociated hippocampal networks on MEAs.

Others studied the nervous systems of intact or semi-intact animals with closed-loop approaches. Nishimura et al. (2013) restored arm movements in a spinal cord-injured non-human primate (NHP) with an artificial cortico-spinal connection and an artificial musculo-spinal connection. This system allows volitional control and boosting of weak, residual muscle activity. Opris et al. (2012) enhanced performance on a delayed match-to-sample task in NHPs using cortical microstimulation contingent on recordings that predict incorrect responses. Dhingra et al. (2013) studied the role of the vagal mechanosensory feedback loop for respiration in a perfused *in situ* brainstem preparation of a mouse model for Rett syndrome. To help map the brain's feedback loops, Beier et al. (2013) demonstrate in the mouse a new transsynaptic retrograde tracer, based on the vesicular stomatitis virus with a rabies virus coat. Egelhaaf et al. (2012) provide a comprehensive review of work on insect vision, emphasizing the importance of active sensing for interpreting optic flow to optimize flying. Ejaz et al. (2013) present closed-loop experiments to study fly visual circuits in which recorded neural responses control a fast turntable on which the fly is mounted. Gollisch and Herz (2012) review how the locust auditory system, salamander retina, and the monkey visual cortex have been used to efficiently explore a large parameter space of iso-response curves, via on-line analysis of incoming data to generate the next stimuli.

The “Model-in-the-loop” paradigm is a powerful approach to understanding complex neural network dynamics. Brookings et al. (2012) interfaced an excised crab stomatogastric ganglion (STG) to a dynamic clamp model neuron to help determine the relative contributions of intrinsic and network properties of STG neurons to network function. Hsiao et al. (2013) interfaced a dentate gyrus-CA1 model to an acute hippocampal slice preparation on an MEA, with the goal of developing cognitive prostheses that could someday replace damaged brain regions.

Theoretical advances are described in several modeling and simulation papers. Witt et al. (2013) modeled the ability of closed-loop optogenetic stimulation to control communication between neural populations by altering their phase relationships. DiMattina and Zhang (2013) reviewed the use of feedback to optimize stimuli continuously during an experiment, for real-time model estimation. Hanuschkin et al. (2013) modeled the sensory-motor loop by which birds learn to produce stereotyped songs. Skocik and Kozhevnikov (2013) demonstrate a system for real-time audio feedback to study birdsong learning. Little and Sommer (2013) optimized exploration strategies in embodied agents based on information-theoretic analysis. Manoonpong et al. (2013) demonstrate the value of adaptive forward models in developing a legged robot locomotion controller. Molkov et al. (2013) modeled the roles of local (brainstem) and distal (lungs) feedback in mammalian respiratory circuits. Wallach (2013) reviews the concept and implementation of a response clamp, in which closed-loop control of a selected neural response variable is used to uncover network properties in cultured networks.

On the clinical side, Afshar et al. (2013) describe and test a new platform for closed-loop deep brain stimulation (DBS). This is the beginning of “smart neuromodulators” that tune themselves to provide optimal benefit to those suffering from, for example, epilepsy or Parkinson's disease. Beverlin and Netoff (2013) present theoretical analysis of a model neural network, aimed at closed-loop seizure control with just such a smart DBS device. Fernandez-Vargas et al. (2013) explored closed-loop optimization of a flickering light display as part of a visually-evoked potential (VEP) brain-computer interface that could be used by locked-in patients to communicate. Walter et al. (2012, 2013) explored transcranial cortical magnetic stimulation (TMS) in a motor task in 3 paralyzed stroke patients wearing a mechatronic hand orthosis. TMS was triggered by recorded brain states that were processed in real time for spectral estimation and to deal with stimulation artifacts.

The diversity of methods, experiments, tools, and analyses in this Research Topic suggests that many more areas of neuroscience research would benefit from adopting a closed-loop perspective.

## Conflict of interest statement

The authors declare that the research was conducted in the absence of any commercial or financial relationships that could be construed as a potential conflict of interest.

## Footnotes

^1^ See: https://mapsengine.google.com/map/edit?mid=zDBeK_5W8FVs.knb4_z5h9NpQ
